# Complications and outcomes after operative treatment of low-energy pelvic ring and acetabular fractures

**DOI:** 10.1007/s00402-026-06418-w

**Published:** 2026-08-03

**Authors:** Kaarina Birgitta Salonpää, Juho Nurkkala, Iikka Lantto, Sanna Kakko, Timo Kaakinen, Mia Aitavaara-Anttila, Janne Liisanantti

**Affiliations:** 1 Oulu University Hospital and Oulu University, Research Unit of Translational Medicine, Oulu, Finland; 2 Helsinki University Hospital and Oulu University, Research Unit of Translational Medicine, Helsinki, Finland

**Keywords:** Pelvic fracture, Low energy, Fragility fracture, Postoperative complications, Traumatic pelvic fracture

## Abstract

**Background:**

Fragility fracture of the pelvis (FFP) with a low-energy mechanism of injury is relatively common among the growing geriatric population. The incidence of postoperative complications after surgically treated FFP is not well documented. The aim of this study was to report complications after operatively treated FFP and to determine whether these complications would have a detrimental effect on outcomes.

**Methods:**

Retrospective study of 200 consecutive patients who underwent operative treatment at Oulu University Hospital, Oulu, Finland, between 1.1.2010 and 15.4.2021 for FFP with a low-energy injury mechanism. Postoperative complications were recorded, and associations with outcomes were depicted.

**Results:**

Postoperative complications were recorded in 31 (15.5%) patients. Patients with complications had higher intraoperative bleeding (OR 2.8 (1.0-7.9), *p* = 0.048) and higher ASA classification 3.4 (1.3–8.4), *P* = 0.009). Patients with complications had longer hospital stays, but no clear association between complications and mortality or discharge location was observed.

**Conclusions:**

The incidence of postoperative complications was comparable with that reported in previous literature. Patients who experienced complications had longer hospital stays; however, no definitive association between complications and outcomes was identified.

## Introduction

Low-energy pelvic fracture or fragility fracture of the pelvic ring (FFP) is a common injury caused by low-energy mechanisms, e.g., a fall from standing height or from a bed. The prevalence of these injuries is rising in the Western world due to the growing share of the geriatric population [1, 2]. FFP injuries lead to decreased quality of life, loss of independence, immobility, and problematic pain [2]. The primary management of FFP is conservative, aiming for adequate pain control and early mobilization [2, 3]. Operative treatment is indicated when unstable fractures prevent initial conservative care or when conservative care fails [1, 2]. The main goal of treatment should always be early, pain-free mobilization and restoration of the patient’s previous self-sufficiency [2].

Trauma surgery in elderly patients carries a substantially elevated risk of both surgical and medical complications due to decreased physiological reserves and frailty [4, 5]. Although the reported complication rate after operative treatment for FFP is relatively modest in previous studies, the current literature lacks specifics on the timing of complications and the factors associated with their occurrence [6, 7].

The purpose of the present study is to examine the rate of postoperative complications following surgical care for FFP, the risk factors for complications, and the impact of complications on outcomes in this patient group.

## Methods

### Patients

This is a retrospective single-center study conducted at Oulu University Hospital (OUH), which provides healthcare to roughly 740,000 inhabitants in northern Finland [Statistics Finland 2025]. All consecutive traumatic low-energy pelvic fractures requiring operative care between 1 January 2010 and 15 April 2021 at OUH were included in the analysis. The World Health Organization criteria, which were utilized in the present study patient enrollment, define a fragility fracture as “a fracture that is caused by an injury that would be insufficient to fracture normal bone; the result of reduced compressive and/or torsional strength of bone.” Patients with malignant fractures were excluded.

The study protocol was approved by the hospital administration (111/2021). Due to the retrospective study design and Finnish legislation, no statement from the local ethics committee was required.

### Data extraction

The patients were identified from the hospital’s patient management system using operation codes NEJ50, NEJ60, NEJ86 & NFB60 and the data were manually extracted from medical records. The collected data included the type of trauma, age, gender, weight, medications, and history of smoking, alcohol abuse, and other substance misuse. The Charlson Comorbidity Index (CCI) was determined, along with diagnoses of dementia and osteoporosis, to reflect an individual’s overall health. Intraoperative blood loss and the need for blood products during the hospital stay were recorded. Blood samples, such as hemoglobin and CRP (C-reactive protein), were collected at admission and on the day of operation. If a patient had another injury (brain injury, an associated long-bone injury, or any soft-tissue injury), it was recorded as an associated injury.

Complications were classified as medical and surgical, following the classification used in our previous report [8]. Postoperative complications were categorized as follows: pneumonia was defined as infiltrates on chest X-ray with leukocytosis and fever, or purulent tracheal secretions. Postoperative atrial fibrillation, acute myocardial infarction, or heart failure were recorded as cardiologic complications. Acute kidney injury (AKI) was defined according to KDIGO (Kidney Disease: Improving Global Outcome) criteria, which define AKI as an increase in serum creatinine by 26.5 µmol/l within 48 h or to ≥ 1.5 times baseline. Acute heart failure was defined as a clinical condition with indicative findings on laboratory tests and/or chest X-ray. Pulmonary embolism and DVT were defined as clinical conditions confirmed by positive imaging findings. Surgical complications (surgical site infection (SSI), hematoma, loss of fixation) were collected from medical records, laboratory results, and radiological reports if they were mentioned and treated. Iatrogenic bleeding was recorded as a complication if it was mentioned in the surgical report. Conservative treatment failure was defined as the patient’s inability to mobilize with or without mobility aids within one month after injury despite adequate analgesia.

Pelvic fractures were classified according to the Rommens Fragility Fractures of the Pelvis (FFP), and acetabular fractures were classified using the Letournel classification. Surgical treatment was performed using standard pelvic and acetabular approaches, including the iliac window corresponding to the first window of the ilioinguinal approach, the Kocher-Langenbeck approach, and the modified Stoppa approach, which serves as an intrapelvic alternative to the third window of the ilioinguinal approach, either alone or in combination. The procedures comprised various forms of open reduction and internal fixation (ORIF) utilizing the aforementioned surgical approaches and sacral screw fixation. Acetabular fractures were treated with ORIF or with total hip arthroplasty (THA) with an enforcement cage and plate. Spinopelvic dissociation due to the sacral fracture was operated on using a posterior approach to the lumbar spine and sacrum (spinopelvic).

### Statistical analysis

IBM SPSS Statistics 29 software (IBM SPSS Statistics for Windows, Version 29.0, Armonk, NY, USA) was used to perform statistical analyses. Categorical variables are reported as numbers (n) and percentages (%), whereas continuous variables are reported as medians and 25–75th percentiles [25–75th PCT]. Categorical variables were tested using Pearson’s Chi-square, and continuous variables were tested using the Mann–Whitney test. Two-tailed P values below 0.05 were considered statistically significant. Logistic regression analysis was performed to calculate the OR for postoperative complications. Age and gender, as well as continuous and categorical variables with univariate significance < 0.1, were included one by one using the enter method. Factors with a P value < 0.05 were kept in the model, as were those with a significant impact on the log-likelihood function (Tables 1 and 2).

## Results

**Table 1:** Rommens classification of 135 FFP and operation type of patients with pelvic fracture

	ORIF (*n* = 4)	ORIF + screws (*n* = 7)	Screws (*n* = 120)	Spinopelvic (*n* = 4)
FFP Ia	0 (0.0)	0 (0.0)	0 (0.0)	0 (0.0)
FFP Ib	1 (25.0)	0 (0.0)	0 (0.0)	0 (0.0)
FFP IIa	0 (0.0)	0 (0.0)	3 (2.5)	0 (0.0)
FFP IIb	0 (0.0)	0 (0.0)	0 (0.0)	0 (0.0)
FFP IIc	0 (0.0)	0 (0.0)	1 (0.8)	0 (0.0)
FFP IIIa	3 (75.0)	1 (14.3)	2 (1.7)	0 (0.0)
FFP IIIb	0 (0.0)	0 (0.0)	1 (0.8)	0 (0.0)
FFP IIIc	0 (0.0)	1 (14.3)	26 (21.7)	0 (0.0)
FFP IVa	0 (0.0)	1 (14.3)	2 (1.7)	0 (0.0)
FFP IVb	0 (0.0)	4 (57.1)	68 (56.7)	3 (75.0)
FFP IVc	0 (0.0)	0 (0.0)	15 (12.5)	1 (25.0)

Values are numbers (percentage)

*FFP* fragility fracture of pelvis, *ORIF* open reduction and internal fixation

**Table 2:** Letournel classification and operation type of 65 acetabulum fracture patients

	ORIF (*n* = 41)	THA (*n* = 24)
AC	3 (7.3)	2 (8.3)
ACPHT	9 (22.0)	5 (20.8)
AW	1 (2.4)	0 (0.0)
BC	18 (43.9)	8 (33.3)
PCPW	1 (2.4)	0 (0.0)
PW	2 (4.9)	1 (4.2)
Roof impact	0 (0.0)	1 (4.2)
T-type	3 (7.3)	5 (20.8)
Transverse	4 (9.8)	2 (8.3)

Values are numbers (percentage)

*ORIF* Open reduction and internal fixation, *THA* Total hip arthroplasty, *AC* Anterior Column, *ACPHT* Anterior Column Posterior Hemitransverse, *AW* Anterior Wall, *BC* Both Column, *PCPW* Posterior Column Posterior Wall, *PW* Posterior Wall, *VS* Vertical Shear, *LC* Lateral Compression

During the study period, there was a total of 200 trauma patients operated on with FFP. A total of 31 (15.5%) patients had at least one postoperative complication. 16 (8.0%) were surgical, and 23 (11.5%) were medical. The most common surgical complication was iatrogenic bleeding: one from the superior gluteal artery during transsacral screw navigation, and the last 5 cases during the anterior approach from the iliac vein or the corona mortis artery. The most common medical complication was pneumonia (Table 3).

**Table 3:** Postoperative complications in 200 trauma patients operated on for FFP

	Number of patients *n* = 200	complication onset (days)
Surgical complications	16 (8.0)	16 [0–20]
Iatrogenic bleeding	6 (3.0)	NA
Postoperative bleeding	2 (1.0)	1,17
Infection	2 (1.0)	14, 20
Reoperation	6 (3.0)	21 [18–28]
Medical complications	23 (11.5)	1 [1–4]
Acute kidney injury	4 (2.0)	1,1,3,3
Pneumonia	13 (6.5)	1 [1–5]
Deep venous thrombosis	0 (0.0)	
Pulmonary embolism	1 (0.5)	2
Acute heart failure	5 (2.5)	1,1,1,1,2

Values are numbers (percentage)

Patients with postoperative complications were more often smokers and had higher ASA classification. Failed conservative treatment attempts were associated with fewer complications, but no statistical difference was observed between surgical timing and complications. Low admission Hb was more common among patients with complications. Higher CRP on the day of operation, larger intraoperative blood loss, and longer operation duration were associated with postoperative complications. Patients with postoperative complications had acetabular fractures more often (21 (67.7) vs. 44 (26.0) < 0.001), whereas no difference in soft tissue injury incidence was observed (Table 4).

**Table 4:** Comparison of patients having postoperative complications

	Patients with postoperative complications *n* = 31	Patients without postoperative complications *n* = 169	*P*-value
Male	14 (45.2)	51 (30.2)	0.143
Age (years)	77 [68–84]	75 [66–82]	0.166
BMI	24.6 [22.2–29.0]	25.6 [22.7–29.3]	0.777
Smoking	9 (29.0)	21 (12.4)	0.027
Alcohol overuse	3 (9.7)	11 (6.5)	0.701
Charlson comorbidity index	5 [4–6]	4 [3–5]	0.081
ASA	3 [3–4]	3 [2–3]	0.025
ASA 4 or 5	9	23	0.031
Days from injury to operation (All patients)	9 [7–20]	16 [5–53]	0.292
Days from injury to operation (not initial conservative treatment try)	9 [6–13]	6 [3–12]	0.136
Failed conservative treatment	6 (19.4)	65 (38.5)	0.019
CRP at the day of operation	48 [26–63]	27 [11–68]	0.040
Admission Hb	110 [101–118]	118 [107–129]	0.005
Admission Hb > 110	14 (45.2)	111 (65.7)	0.027
Blood products before operation	1	10	0.569
Blood loss during surgery (milliliters)	400 [20–700]	8 [0-450]	0.016
Blood loss>200 ml	12 (38.7)	39 (23.1)	0.028
Admission AIS score	3 [2–3]	2 [2–3]	0.011
Admission ISS score	9 [7–9]	4 [4–9]	0.009
ISS > 15	1 (3.2)	9 (5.3)	0.609
Operation duration (min)	137 [70–177]	54 [37–134]	< 0.001
Operation duration over 120 min	19 (61.3)	46 (27.2)	< 0.001
Acetabulum fracture	21 (67.7)	44 (26.0)	< 0.001
Soft tissue injury	0 (0.0)	3 (1.8)	0.453
Associated injury	4 (12.9)	18 (10.7)	0.713

Values are numbers (percentage) or medians (25–75th percentiles)

*BMI* Body mass index, *ASA* American society of anesthesiologists score, *CRP* C-reactive protein, *Hb* Hemoglobin, *AIS* Abbreviated injury scale, *ISS* Injury severity score

Logistic regression analysis showed that ASA classification and intraoperative blood loss > 200 mL were independently associated with postoperative complications (Table 5).

**Table 5:** Logistic regression analysis for postoperative complications

	OR and 95% CI	*p*-value
ISS	0.9 (0.8–1.1)	0.481
ASA	3.4 (1.3–8.4)	0.009
Intraoperative blood loss>200 ml	2.8 (1.0-7.9)	0.048

*OR* Odds ratio, *CI* Confidence interval, *ISS* Injury severity score, *ASA* American Society of Anesthesiologists score

Patients with postoperative complications were more likely to require ICU admission and had longer hospital stays. Three patients with postoperative complications died during their hospital stay. Survival time was shorter, and 5-year mortality was higher among patients with complications. Patients who died postoperatively within 5 years were significantly older than 5-year survivors (85 [62–88] vs. 76 [67–84]). No difference in discharge location was observed in this data (Table 6).

**Table 6:** Outcomes

	Patients with postoperative complications *n* = 31	Patients without postoperative complications *n* = 169	*P*-value
ICU admission	8 (25.8)	7 (4.1)	< 0.001
Hospital LOS	7 [4–11]	5 [3–8]	0.020
In-hospital death	3 (9.7)	0 (0.0)	< 0.001
Survival time (days)	667 [133–1602]	1276 [549–2166]	0.025
5-year mortality	10 (32.3)	23 (13.6)	0.010
Discharged home	4 (12.9)	33 (19.5)	0.383
Discharged to the regional health care center’s ward	13 (41.9)	96 (56.8)	0.267
Discharged to regional hospital	11 (35.5)	40 (23.7)	0.165

Values are numbers (percentage) or medians (25–75th percentiles)

*ICU* Intensive care unit, *LOS* Length of stay

Acetabular fracture patients who experienced postoperative complications were older and had slightly higher Charlson comorbidity index scores. Recorded AIS and ISS scores were comparable between groups, and no difference in discharge locations was observed (Table 7).

**Table 7:** Preoperative characteristics and outcomes of 65 patients suffering acetabular fractures

	Patients with postoperative complications *n* = 21	Patients without postoperative complications *n* = 44	*P*-value
Male	13 (61.9)	28 (63.6)	0.892
Age	78 [69–85]	73 [67–82]	0.109
Charlson comorbidity index	4 [4–7]	4 [3–5]	0.123
Admission AIS score	3 [3–3]	3 [3–3]	0.566
Admission ISS score	9 [9–9]	9 [9–9]	0.566
In-hospital death	2 (9.5)	0 (0.0)	0.038
Discharged home	2 (9.5)	4 (9.1)	0.955
Discharged to the regional health care center’s ward	8 (38.1)	25 (56.8)	0.158
Discharged to regional hospital	9 (42.9)	15 (34.1)	0.493

Values are numbers (percentage) or medians (25–75th percentiles)

*AIS* Abbreviated injury scale, *ISS* Injury severity score

## Discussion

This study included 200 FFP with a low-energy trauma mechanism. A total of 31 patients (15.5%) had 39 recorded complications, of which 16 were classified as surgical and 23 as medical. The most common surgical complication was iatrogenic bleeding, and the most common medical complication was pneumonia. In univariate analysis, injury severity correlated with complication occurrence. In logistic regression analysis, an elevated ASA score and larger intraoperative bleeding were associated with postoperative complications. Patients with complications were more often admitted to the ICU, and their hospital LOS (Length of stay) was longer. Five-year mortality remained elevated among patients with complications, but the observed difference was mostly explained by the deceased patients’ older age.

Injury severity seemed to correlate with the occurrence of postoperative complications in this dataset, despite a low-energy trauma mechanism. This finding has been previously reported in a combined large dataset of conservatively and operatively treated geriatric patients with pelvic fracture [2]. Patients who developed complications had higher initial ISS and AIS scores, more often had acetabular fractures, had lower admission Hb levels, and had larger intraoperative blood loss. Notably, a primary conservative treatment attempt was more often recorded among patients without complications. Patients with primary conservative treatment most likely had less severe injuries that were initially deemed to heal without operation, supporting the assumption that injury severity correlates with complications. In a recent study by Romens et al., the researchers found that the postoperative complication rate following operative treatment for FFP was not significantly affected by the type of procedure. They discussed that prolonged in-hospital LOS itself, rather than the type of procedure, could have predisposed to complications [7]. The presented hypothesis would align with our finding, indicating that injury severity predisposes to complications after FFP, since patients with more severe injuries tend to have longer hospital stays [2]. When patients with acetabular fractures were examined separately, injury severity measured by AIS and ISS scores showed no clear association with complications. No difference in discharge locations was observed among acetabular fracture patients with or without complications.

In this study, an association between ASA classification and postoperative complications was observed, consistent with prior orthopedic literature [4, 5, 9]. Interestingly, elevated ASA classification was a stronger predictor of complications than the CCI score in this cohort, despite the CCI’s demonstrated association with postoperative complications in perioperative settings [5, 9]. The present cohort’s overall high median age may have attenuated the CCI’s association with complications because age is a variable in the CCI scoring system, unlike the ASA classification. Intraoperative bleeding was associated with postoperative complications in our cohort. Blood transfusions and major intraoperative bleeding are known risk factors for postoperative complications during hip fracture surgery [10, 11]. In the present cohort, the limited cutoff of blood loss > 200 mL was associated with postoperative complications, emphasizing diminished physiological reserves and frailty in this patient group. Notably, despite the observed statistical and clinical difference in intraoperative bleeding, the actual measured volumes were relatively modest in both groups.

Patients with postoperative complications were more likely to be admitted to the ICU, and their hospital LOS was longer, findings that were expected [2]. In-hospital mortality was low; only three patients with complications died during their hospital stay, consistent with prior literature [1]. Five-year mortality was statistically higher among patients with postoperative complications. However, 5-year survivors were distinctly younger than non-survivors; a nine-year difference in median age obviously has a significant impact on long-term mortality in patients aged above 75 years. Despite longer in-hospital LOS and morbidity, postoperative complications had no impact on discharge location in this study. Less than 20% of patients were discharged directly home, whereas the rest were enrolled in a regional hospital or a regional health care center’s ward. Similar results were obtained regardless of treatment type (conservative or operative) among geriatric FFP patients [7, 12]. In a recent nationwide cohort study in the United States, more than 70% of geriatric FFP patients treated conservatively or operatively were discharged to long-term care facilities from the hospital, underscoring the overall postoperative challenges in this fragile patient group regarding outcomes following FFP [].

### Limitation

This study has some limitations. Although the cohort was harvested from a relatively long period, the actual cohort size was somewhat modest. This limits the ability to observe possible associations with specific trauma types and outcomes. The retrospective study design introduces limitations when interpreting potential causal relationships. We used logistic regression analysis to improve the quality of our results to mitigate this issue. Data on long-term outcomes (e.g., daily functioning, quality of life) were unfortunately lacking, which is a limitation.

## Conclusions

Fragility fractures of the pelvis pose a major challenge for the growing number of geriatric patients. The present cohort’s postoperative complication rate was modest and slightly lower than in preceding large reports with mixed patient cohorts [1, 2]. Baseline physiologic status and the severity of the injury mechanism appear to be precursors to postoperative complications among operatively treated FFP patients. Based on our findings, postoperative complications were associated with prolonged LOS, whereas no detrimental effect on discharge location or long-term mortality was observed. A minority of patients were discharged directly to home.

## Data Availability

No datasets were generated or analysed during the current study.
